# Bioadhesive Mini-Tablets for Vaginal Drug Delivery

**DOI:** 10.3390/pharmaceutics6030494

**Published:** 2014-08-27

**Authors:** Marianne Hiorth, Susanne Nilsen, Ingunn Tho

**Affiliations:** 1 SiteDel Research Group, School of Pharmacy, Faculty of Mathematics and Natural Sciences, University of Oslo, 0316 Oslo, Norway; E-Mail: marianne.hiorth@farmasi.uio.no; 2 Drug Transport and Delivery Research Group, Department of Pharmacy, Faculty of Health Sciences, University of Tromsø, 9037 Tromsø, Norway; E-Mail: susanils@hotmail.com

**Keywords:** bioadhesive polymers, vaginal application, multi-particulates, non-ionic cellulose ethers, hexyl aminolevulinat hydrochloridum

## Abstract

Different non-ionic cellulose ethers (methyl cellulose, MC; hydroxyethyl cellulose, HEC; hydroxypropyl cellulose, HPC; hydroxypropylmethyl cellulose, HPMC) and microcrystalline cellulose (MCC) were investigated as matrix formers for preparation of mini-tablets targeting vaginal drug delivery. Hexyl aminolevulinat hydrochloridum (HAL) was used as a model drug. The mini-tablets were characterized with respect to their mechanical strength, bioadhesion towards cow vaginal tissue in two independent tests (rotating cylinder test, detachment test using texture analyzer), and dissolution rate in two media mimicking the pH levels of fertile, healthy and post-menopausal women (vaginal fluid simulant pH 4.5, phosphate buffer pH 6.8). Mini-tablets with a matrix of either HPMC or HPC were found to possess adequate mechanical strength, superior bioadhesive behavior towards vaginal tissue, and pH independent controlled release of the model drug, suggesting that both systems would be suited for the treatment of women regardless of age, *i.e.*, respective of their vaginal pH levels. Bioadhesive mini-tablets offer a potential for improved residence time in the vaginal cavity targeting contact with mucosal tissue and prolonged release of the drug.

## 1. Introduction

The vagina is an important application site for drug delivery, especially for local therapy of different diseases, such as bacterial, fungal and protozoa infections, for HIV prevention, delivery of contraceptives, spermicides or labor-inducers and for the treatment of precancerous lesions [1,2]. It may also serve an alternative route for systemic drug delivery [3].

Although the vaginal tissue is referred to as mucosal, the vagina does not have secretory glands. However, a mixture of fluids originating from a number of different sources comprises a moist film coating the vaginal surface. The pH of healthy pre-menopausal women of 3.5–4.5 is provided by lactic acid produced by the bacteria Lactobacillus, an essential part of the vaginal microflora [3,4]. The composition, volume, pH and viscosity of the vaginal fluids are affected by age, cyclic hormone changes, and sexual activity; factors that may also influence the effect of vaginally applied drug delivery systems or dosage forms. Changes due to infections or pregnancy may also lead to different activity of drugs [3].

In order to maintain high patient compliance and adherence to therapy, the dosage forms or delivery systems should also be easy to administer and not cause discomfort or irritation. It should provide high efficiency based on an even distribution and long retention time of the drug in the vagina [3]. Different types of conventional dosage form, such as creams, gels, ointments and tablets, have been investigated for vaginal drug delivery, most of which has major drawback, and have been described as messy, uncomfortable, leaking in the underpants *etc.*, resulting in low compliance. The low retention time is a well-known problem encountered in the formulation of drugs for vaginal application, and can also be attributed to the self-cleansing action of the vaginal tract [5,6]. Bioadhesive polymers are often included in the formulation to increase the retention time on the mucosal tissue [5,7]. The most widely investigated group of bioadhesive polymers are hydrophilic polymers containing numerous hydrogen bond forming groups, such as carbomers, chitosan, sodium aginate and cellulose derivatives [7]. Being water-soluble, the polymers become adhesive on exposure to moisture, and will readily cohere to surfaces. They are known to produce high viscosity at low concentrations, but most of the polymers are pH sensitive and may therefore behave differently depending on the vaginal pH.

Lately, nanopharmaceuticals, e.g., liposomes, dendrimers, cyclodextrines and other nanoparticles, have gained attention also for vaginal delivery [6,8]. However, a major disadvantage of most nanopharmaceuticals is their liquid nature and their consequently low residence time within the vagina. Therefore, different types of vehicle have been proposed to prolong the retention time. Advanced delivery systems, such as liposomes-in-gels, have shown promising characteristics in *in vitro* and *ex vivo* experiments (e.g., [9,10]). Among advanced new systems from the research front that has entered into clinical trials can be mentioned: VivaGel^®^ (Starpharma, Melbourne, Australia), which is a dendrimer-based antimicrobial platform targeting a range of sexual health products. VivaGel^®^ is based on a Carbopol gel with acidic buffering capacity and mucoadhesive properties containing polylysine dendrimers that binds to bacteria or viruses and prevents them from affecting the organism’s cells. The VivaGel^®^ bacterial vaginosis (BV) has been confirmed to cure signs and symptoms of BV in Phase III clinical trials, and is currently undergoing studies to investigate the effectiveness in preventing reoccurrence of BV. The VivagGel^®^ platform is also an interesting technology for prevention of transmission of genital herpes, HIV and other sexual transmitted infections including human papillomavirus (HPV), the causative agent of cervical cancer. Other examples of bioadhesive gel formulations are Carraguard^®^, a carrageenan based gel that has been shown to prevent transmission of high-risk HPV, but not HIV, in clinical trials by acting as an attachment inhibitor at the vaginal epithelium. An example of a bioadhesive gel which is on the market is Glynol II^®^ Contraceptive Jelly, a spermicide containing gel (nonxynol-9) based on a combination of sodium carboxmethylcellulose and polyvinylpyrrolidone. Another promising approach is the foam technology platform from Foamix, targeting vaginal among other topical applications. The vaginal foam is bioadhesive emollient foam based on an *o*/*w* emulsion with a bioadhesive polymer. The company has different vaginal foams in the pipeline containing acyclovir, imiquimod and estradiol targeting herpes genitalis, genital warts and atrophic vaginitis, respectively.

Solid formulations have the advantage of high dose accuracy and long term stability, as compared to semi-solid systems. However, the vaginal disintegration of conventional vaginal tablets is often slow, and the tablets are often rapidly cleared due to gravity combined with the self-cleansing action of the vagina. This may be circumvented by use of bioadhesive polymers in the formulation [11,12,13], but some studies also reported loss of bioadhesive tablets [11]. Multiparticulate systems (e.g., pellets or mini-tablets) can be used to overcome the problems associated with monolithic systems; the dose is divided into multiple smaller units that will spread out more in the vaginal cavity and contribute to improved coverage of the vaginal epithelium. Also, the loss of a few multiparticulates will have less impact of the outcome of the treatment. Bioadhesive multiparticulates are expected to swell and form micro-gels, releasing the drug in a controlled manner and maximizing the drug availability at the delivery site. Another multiparticulate approach for vaginal delivery is the microcrystalline starch pellets, which disintegrates in the vaginal cavity [14,15]. The pellets show fast disintegration also *in vivo* resulting in even distribution on the vaginal wall and prolonged retention of the formulation in the human vaginal cavity [16,17,18,19]. An alternative to the conventional solid dosage forms is intravaginal rings; e.g., a newly developed ring containing both an antiretroviral drug and a contraceptive has just entered into clinical trials [20].

The aim of the current study was to investigate bioadhesive mini-tablets for vaginal drug delivery. Mini-tablets are tablets with a diameter of 1–3 mm [21,22]. Hexyl aminolevulinat hydrochloridum (HAL) was chosen as a model drug because of its potential application in photodynamic therapy (PDT) of topical cancers, such as cervical cancer [2,23]. In PDT, a photoreactive substance (photosensitizer) is activated by illumination with light of a certain wavelength resulting in formation of reactive oxygen species, which will kill the cancer cells [24]. HAL is a derivative of the endogenous substance 5-aminolevulinic acid (ALA), a precursor of the endogenous photosensitizer protoporphyrin IX (PpIX). Exogenous administration of 5-ALA, or its derivatives, induces accumulation of PpIX in cancerous lesions [25]. HAL is the hexyl-ester of 5-ALA. For clinical studies of cervical intraepithelial neoplasia (CIN), doses of 10 mL of 10 mM HAL-thermogel applied topically 5–9 h before photodynamic therapy have been reported [23,25]. HAL has a pKa of 8.31 for the amino-group and is freely water-soluble (0.8 g in 1 g), but unstable in a moist environment [26,27]. Therefore, to eliminate the stability issues encountered with moist production steps, we propose direct compression as a suitable method for preparation of HAL mini-tablets. Non-ionic cellulose ethers were chosen as matrix formers since they are not influenced by changes in pH of the environment, and should therefore be expected to show the same performance in pre- and post-menopausal women. Bioadhesive properties were targeted to further prolong the retention of the mini-tablets on the vaginal mucosa.

## 2. Experimental Section

### 2.1. Materials

Hexyl aminolevulinat hydrochloridum (HAL) was kindly provided by Photocure ASA, Oslo, Norway. Hypromellose (Hydroxypropylmethyl cellulose, HPMC) (Metolose 90SH-4000) was from Shin-Etsu Chemical Co, Tokyo, Japan, and hyprolose (hydroxypropyl cellulose, HPC) (Klucel^®^ HF Pharm), hydroxyethyl cellulose (HEC) (Natrosol^®^ 250LR) and methylcellulose (MC) (Culminal^®^ MC-2000) were products of Hercules—Aqualon, Wilmington, Germany. Microcrystalline cellulose (MCC) (Avicel^®^ PH101) was obtained from FMC biopolymers, Leeds, UK. Table 1 provides an overview of the polymers.

**Table 1:** Overview of cellulose derivatives used in the current study.

Polymer	Substituent Group	Molecular Weight (Dalton) *
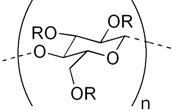		
**Microcrystalline cellulose (MCC)**	R = H	<37,500 **
**Methyl cellulose (MC)**	R = H or CH_3_	70,000
**Hydroxyethyl cellulose (HEC)**	R = H or CH_2_CH_2_OH	90,000
**Hydroxypropyl cellulose (HPC)**	R = H or CH_2_CH(OH)CH_3_	1,150,000
**Hydroxypropylmethyl cellulose (HPMC)**	R = H or CH_3_ or CH_2_CH(OH)CH_3_	1,200,000

* Typical values according to product information; ** Estimated from degree of polymerization; not more than 350 according to European Pharmacopeia (Ph.Eur).

### 2.2. Test Media

Vaginal fluid simulant (VFS) was prepared by modification of the composition originally reported by Owen and Katz [3]. It contained 3.51 g/L NaCl, 1.40 g/L KOH, 0.222 g/L Ca(OH)_2_, 2 g/L lactic acid, 1 g/L acetic acid, 0.16 g/L glycerol, 0.4 g/L urea and 5 g/L glucose. The pH of the mixture was adjusted to 4.5 with either HCl or NaOH.

Acetate buffer pH 4.5 was made by dissolving 77.1 g ammonium acetate in in distilled water. Seventy milliliter of glacial acetic acid was added and the mixture was diluted to 1000 mL with distilled water.

Phosphate buffer pH 6.8 was made from 51.0 mL 0.2 M KH_2_PO_4_ and 49.0 mL of 0.5 M Na_2_HPO_4_ diluted to 1000 mL with distilled water. The pH was adjusted to 6.8 with either HCl or NaOH when necessary.

### 2.3. Particle Density

The true density of the polymers was determined using a helium gas pycnometer (AccuPyc 1330, Micromeritics Instrument Corporation, Norcross, GA, USA). Reported results were the mean of two independent experiments with 10 repetitive purge cycles and three runs for each experiment.

### 2.4. Preparation of Tablets

HAL was mixed separately with each of the polymers (MCC, HC, HEC, HPMC and HPC) in the ratio 10:90 (*w*/*w*) by hand, and compressed to mini-tablets on a costume-made compaction simulator (ServoPress 450, Schmidt Technology GmbH, St. Georgen, Germany, with the compaction module, IBR, Waldkirch, Germany) equipped with 2 mm concave 15-tip multiple tooling (Ritter Pharma–Technik GmbH, Stapelfeld, Germany). Prior to compression, the tips were lubricated using a magnesium stearate in acetone suspension. The mini-tablets were prepared by manual die-filling. The compaction speed was 10 mm/s. Compaction pressure was 150 ± 5 MPa. The filling volume was kept constant; hence, the mass of the biconvex mini-tablet varied depending on the density of the powder mixture.

In addition, 6 mm flat-faced tablets were prepared following the same procedure, for use in the detachment test.

For HPMC and HPC, additional formulations containing HAL to polymer in the ratio 1:99 and 50:50 (*w*/*w*) were prepared both as 2 mm mini-tablets and 6 mm tablets.

### 2.5. Mechanical Strength of the Mini-Tablets

Thirty mini-tablets of each batch were evaluated with respect to height and crushing force using a texture analyzer (TA-XT2i Stable Micro Systems, Godalming, UK) with a 50 kg load cell at ambient temperature. For height measurements, the instrument was calibrated using calibration blocks DIN 861 (W&Z Computer Vertrieb GmbH, Dresden, Germany) with the respective size of 1.000, 1.300, 1.400, 1.500, 1.600, 1.700, 1.800, 1.900, 2.000, 2.100 and 3.000 mm. A calibration equation was derived, which was used for correction of the measured heights. The axial height of mini-tablets was determined using a speed of 0.10 mm/s and a trigger force of 5 g. The height registered at detection of the trigger force was taken as the mini-tablet height and corrected using the calibration equation.

The diametrical force needed to crush the mini-tablet was determined at a speed of 0.3 mm/s applying a trigger force of 5 g. The tensile strength was calculated using the equation for flat-faced tablets according to Fell and Newton [28]. Even though min-tablets are biconvex, this approximation has been used in literature for estimation of tensile strength of mini-tablets [21,22].

### 2.6. Ex Vivo Assessment of Bioadhesion

#### 2.6.1. Preparation of Tissue

Cow vaginal mucosa was chosen as a biological matrix, since it is recognised as suitable for the simulation of human vaginal mucosa properties [10,12,29]. Vaginal mucosal tissue from heifer was obtained from the slaughterhouse (Mydland, Tromsø, Norway). The tissue was collected fresh, immediately after slaughtering, and transported in acetate buffer pH 4.5 on ice to the laboratory, where the vaginal mucosa was carefully removed from the underlying tissue and cleaned with acetate buffer pH 4.5. The tissue was cut into smaller pieces and packed in plastic (cling film) and aluminium foil before freezing at −20 °C. Prior to testing, the tissue was defrosted in acetate buffer pH 4.5 at 37 ± 1 °C for 60 min using a magnetic stirrer and heated disc [29]. The tissue was cut into desirable-sized pieces and applied in the tests of bioadhesion.

#### 2.6.2. Rotating Cylinder Method

The bioadhesion of mini-tablets to vaginal tissue was assessed in a modified version of the rotating cylinder method [30] as previously described [27]. Briefly, the vaginal tissue was attached to a cylinder, and 10 mini-tablets from the same batch were gently placed on the tissue without application of force. The cylinder with the tissue was placed in a chamber containing 400 mL acetate buffer pH 4.5 at 37 °C, and rotated at 150 rpm for 5 min. The number of mini-tablets remaining on the tissue after agitation was counted. The test was run in triplicate, and the results were given in percentage attached after agitation.

#### 2.6.3. Detachment Test

The bioadhesiveness was studied further using in a texture analyzer (TA-XT2i Stable Micro Systems, Godalming, UK) with a 50 N load cell equipped with a mucoadhesion rig. The tablet was attached to a flat-faces probe using double-sided adhesive tape. Due to the small contact area of biconvex 2 mm mini-tablets, it was not possible to obtain appropriate results; therefore, 6 mm flat-faced tablets (prepared as described for mini-tablets) were employed in this test. The vaginal tissue (approximately 20 × 20 mm) was mounted in the holder and hydrated with 100 µL acetate buffer pH 4.5 (ambient temperature). After 1 min, the probe with the attached tablet was moved down to contact the tissue. The contact time between the tablet and the tissue was 30 s applying a force of 5.0 g. The probe was subsequently withdrawn at a constant speed of 0.1 mm/s. The maximum force registered in the detachment test was taken as the detachment force (*F*_max_) and the area under the curve (AUC) of the detachment force *versus* displacement was taken as the work of adhesion [31]. The test was run in eight parallels for each formulation. For reliable measurements, it is important that the registered detachment is in fact the detachment of the tablet from the tissue and not e.g., splitting of the tablet (due to capping/layering) or detachment of the tablet from the probe (adhesive tape), none of which were a problem in the current tests.

### 2.7. Dissolution Rate of Hexyl Aminolevulinat Hydrochloridum (HAL) from the Mini-Tablets

Samples of approximately 400 mg (80 mini-tablets) were tested in 400 mL test medium using the paddle apparatus at 100 rpm and 37 ± 0.5 °C. As test media, vaginal fluid simulant pH 4.5 and phosphate buffer pH 6.8 were used. Samples of 2.0 mL were withdrawn after 5, 10, 15, 30, 45, 60 and 120 min, respectively. The samples were filtered using 0.22 μm syringe filter (Merck Millipore, Darmstadt, Germany), and immediately quantified by HPLC–UV. The HPLC (Waters, Milford, CT, USA) method was based on reversed phase using a C8 column with 0.02 M phosphate buffer and methanol as the mobile phase (40/60), isocratic elution and UV-detection (Waters 2489 UV–vis detector) at 210 nm. The flow rate, injection volume and column temperature was set at 1.5 mL/min, 50 µL and ambient temperature, respectively. HAL was quantified using external standard calibration (peak area). The method was validated with respect to precision as repeatability (RSD ≤ 2%), accuracy (60%–70%) and linearity (*r* = 0.992, range 70%–130% of target concentration). Results were calculated as mean (*n* = 3), and the variation expressed as highest and lowest deviation from the mean.

### 2.8. Statistics

Statistical significance of difference between formulations was determined using two-tailed Students *t*-test. *p* < 0.05 was considered statically significant.

## 3. Results and Discussion

The proper vaginal formulation should spread well out in the vaginal cavity to obtain good effect and have bioadhesive properties to ensure sufficiently long retention time at the delivery site and maximize drug activity. Previously, we have investigated multiparticulates of HAL prepared by extrusion/spheronization of Carbopol 934-containing blends [27]. Since HAL is sensitive to degradation in contact with moisture, the wet-massing step of the preparation process, and the remaining moisture content of the products resulted in poor storage stability of the formulations. By preparation of mini-tablets by direct compression, this issue is avoided.

### 3.1. Preparation of Mini-Tablets

Mini-tablets were prepared from all the different polymers in a drug to polymer ratio of 10:90 (*w*/*w*) (Table 2). The filling volume was kept constant for all formulations; since the polymers have different density, the mass of the mini-tablets varied between the formulations, with MCC producing the heaviest and HPC the least heavy units.

The tensile strength of the mini-tablets showed varying mechanical strength, with those prepared with MCC being the strongest, followed by formulations with HPMC and HPC, respectively (Table 2). Mini-tablets prepared from MC and HEC possessed low mechanical strength. The strength of the HEC mini-tablets was border line with what is allowable with careful handling without falling apart. The mechanical strength reflects the formulation’s ability to form tablets by direct compression under the applied pressure (150 ± 5 MPa). The excipients producing the strongest tablets (MCC, HPC and HPMC) are frequently used as binders in direct compression and/or dry granulation [32], and therefore expected to produce strong tablets. MC and HEC may also be used as binder in tablet formulation, but more in wet granulation, which could explain why the mechanical strength of the directly compressed mini-tablets was lower with these excipients.

**Table 2:** Overview of particle density of the polymers and tensile strength of 2 mm biconvex mini-tablets consisting of 10% (*w*/*w*) hexyl aminolevulinat hydrochloridum (HAL) and 90% (*w*/*w*) of the respective polymers.

Polymer	Particle Density * [g/cm^3^]	Tensile Strength ** [N/mm^2^]
Microcrystalline cellulose (MCC)	1.518 ± 0.008	3.09 ± 0.55
Methyl cellulose (MC)	1.319 ± 0.002	0.46 ± 0.13
Hydroxyethyl cellulose (HEC)	1.334 ± 0.001	0.13 ± 0.02
Hydroxypropyl cellulose (HPC)	1.203 ± 0.001	1.30 ± 0.37
Hydroxypropylmethyl cellulose (HPMC)	1.341 ± 0.001	1.42 ± 0.45

* *n* = 6; ** *n* = 30.

### 3.2. Bioadhesive Characteristics

The two mechanically weak formulations, namely mini-tablets based on MC and HEC, were found to disintegrate immediately upon exposure to fluids. Thus, no results could be obtained with respect to their bioadhesive behaviour towards vaginal tissue.

**Figure 1:**
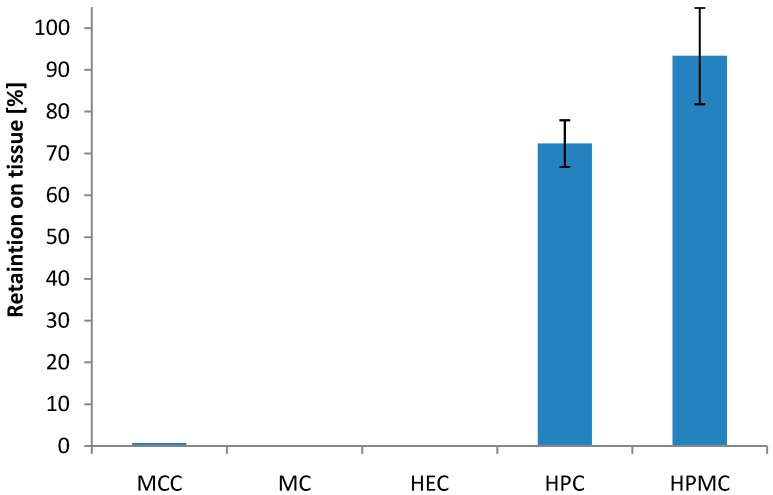
Effect of the type of polymer on the bioadhesion of 2 mm biconvex mini-tablets to vaginal tissue represented as percentage of retained units on the tissue after applied stress in the rotating cylinder test; drug to polymer ratio 10:90 (*w*/*w*); (*n* = 30). Mini-tablets of methyl cellulose (MC) and hydroxyethyl cellulose (HEC) disintegrated and no result was obtained.

In the rotating cylinder test, none of the mini-tablets made of MCC were retained on the tissue after agitation (Figure 1). Mini-tablets containing HPC and HPMC showed high degree of retention on the vaginal tissue after agitation. More than 72% was retained for the HPC-based formulations and more than 93% was retained for the HPMC-based formulations. This is in the same order of magnitude as found for the most bioadhesive Carbopol 934-containing pellets in the previous study [27]. The rotating cylinder test showed a significant difference in bioadhesive behaviour between mini-tablets based on HPC and HPMC (*p* < 0.05). Furthermore, mini-tablets of HPMC also seemed to swell more, or more precisely increase more in size during swelling, as compared to those consisting of HPC. It has been shown in general terms that the swelling state of the polymer contributes to its bioadhesive characteristics [33]. The less hydrophobic character of HPMC compared to HPC may contribute to a higher swelling and hence the higher extent of bioadhesion observed for these mini-tablets towards the vaginal tissue. Also, the drug itself may contribute to the bioadhesive nature of the formulations; HAL is positively charged at pH 4.5 (pKa 8.31) and may therefore interact with the negatively charged tissue surface and with mucin. For HPMC and HPC formulations, it may seem like a correlation between retention of the mini-tablets on the tissue and their respective tensile strength; however, the highest tensile strength was found for the mini-tablet formulation with MCC, which did not show any retention to the tissue in the rotating cylinder method.

**Figure 2:**
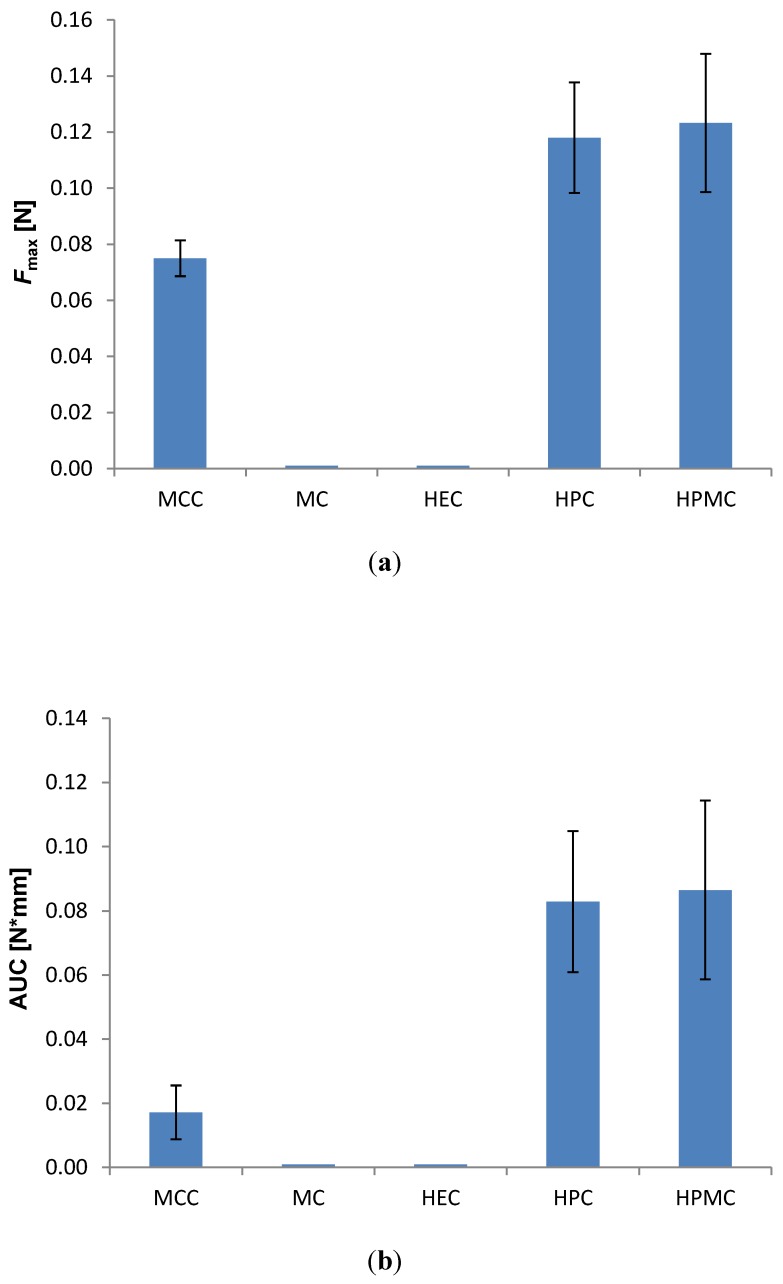
Effect of the type of polymer on the bioadhesion of 6 mm flat-faced tablets to vaginal tissue in the detachment test; drug to polymer ratio 10:90 (*w*/*w*); (*n* = 8). (**a**) Max detachment force (*F*_max_); (**b**) Work of adhesion (AUC). Tablets of MC and HEC disintegrated and no result was obtained.

To further quantify the bioadhesive character of the mini-tablets, the detachment test was employed. However, due to the biconvex shape of the mini-tablets and the small contact area both with the probe (double-sided tape) and the tissue, the mini-tablets detached more often from the probe than it detached from the vaginal tissue, and no reproducible measurements could be obtained. Therefore, to allow measurements, larger fat-faced tablets of 6 mm diameter were prepared with the same compositions as the mini-tablets. These allowed quantification of the max detachment force (Figure 2a) and the work of adhesion (Figure 2b).

Even though the amount of fluid present during measurements was considerably lower in this test, the MC and HEC tablets started disintegrating upon contact with fluids, and no results were obtained for the two formulations. The highest detachment force and the highest work of adhesion were found for HPC and HPMC (Figure 2). The order of magnitude for the detachment force was in the lower region of what has been reported in a similar test for much larger tablets (13 mm diameter) composed of blends of HPMC and Carbopol [12]. Since the tablets in our study are significantly smaller (6 mm diameter), the contact area towards tissue is also smaller. In our study, no significant difference was observed in bioadhesion between the two polymers HPC and HPMC, but they showed significantly higher values in both detachment force and work of adhesion than MCC.

MCC is generally not considered as a bioadhesive polymer, although it swells to a certain degree in water and has several hydrogen bond forming groups. The unexpectedly high detachment forces determined in the current study might be a result of interfering capillary forces, even though care was taken to avoid the influence of capillary forces on the measurements; prior to each test, both the tablet and tissue were well hydrated with acetate buffer pH 4.5. A well-hydrated tissue was chosen to better mimic the moisture-film coating the tissue of the vaginal cavity, as an excess of fluid is less biologically relevant. The production of vaginal fluid is reported to be around 6 g daily with approximately 0.5–0.75 g present in the vagina at any time [4]. The fact that the work of adhesion is relatively much lower for MCC-tablets as compared to the two other polymers supports the hypothesis of influencing capillary forces. It is less likely that capillary forces play a role in the case of the true bioadhesive polymers HPC and HPMC, as the surface layer swells quickly in contact with test medium and forms a gel-like surface. Also, the positive charge of HAL at this pH might contribute to the unexpectedly high detachment force observed for the MCC-formulations.

Comparing the results obtained in the two bioadhesion tests, the main trends were similar. The rotating cylinder method differentiated between the bioadhesive capacity of HPC and HPMC, whereas the detachment did not. This is probably attributed to the different test conditions rather than the properties of the polymers. Retrospectively, the detachment force registered for MCC suggests that capillary forces might have influenced this particular result. In the literature, a variety of tests are used to assess bio- and mucoadhesiveness [34]. The two tests applied in the current study are among the most commonly used. Interestingly, as pointed out in the review of Woertz *et al.* [34], even though the European Pharmacopeia monograph on oromucosal preparations contains a chapter on mucoadhesive preparations, it lacks an official standardized test for these preparations [35].

### 3.3. Dissolution Behavior

**Figure 3:**
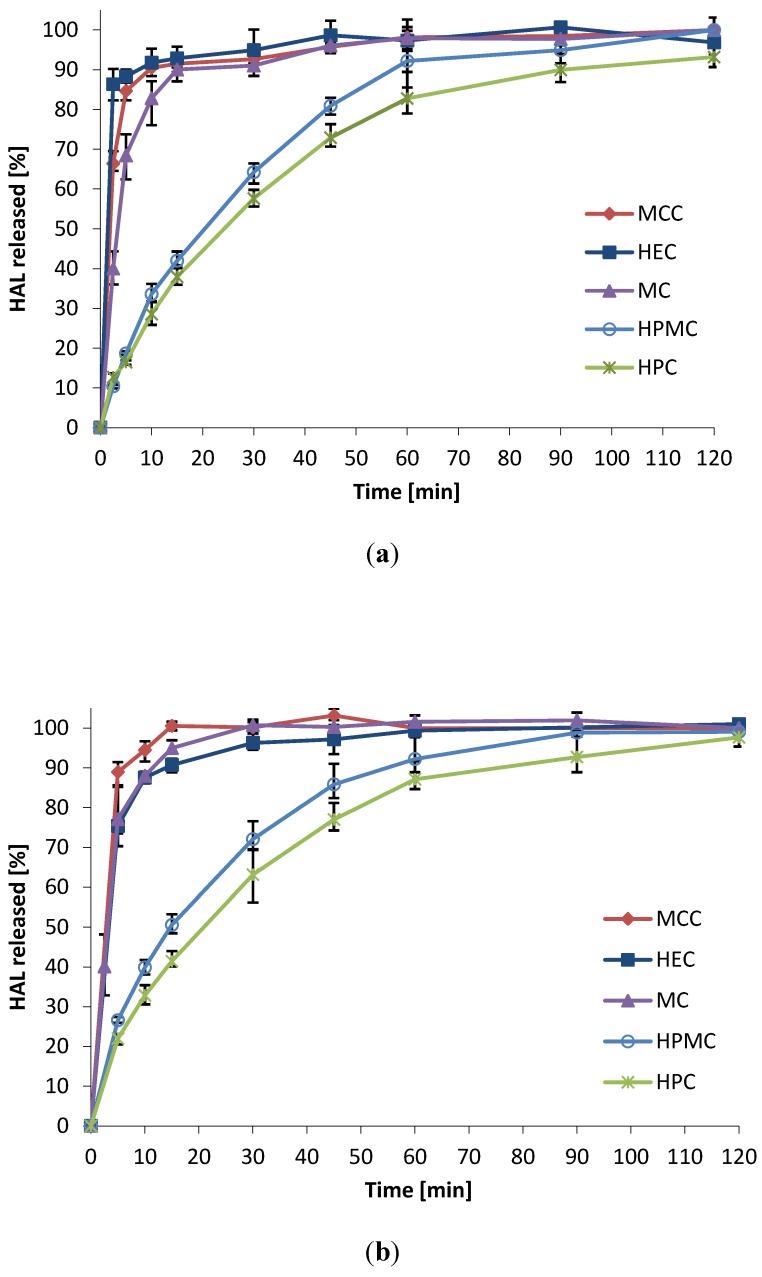
Dissolution rate of HAL from 2 mm convex mini-tablets in two media; drug to polymer ratio 10:90 (*w*/*w*); (*n* = 3) (**a**) Vaginal fluid simulant pH 4.5; (**b**) Phosphate buffer pH 6.8.

The drug release from the mini-tablets was evaluated in two different media mimicking the pH of pre- and post-menopausal women. Vaginal fluid simulant (VFS) pH 4.5 was used to simulate the conditions of vaginal fluids in healthy, fertile women [4], whereas a more simple phosphate buffer pH 6.8 simulated the higher pH conditions post menopause [36]. Mini-tablets based on MCC, MC and HEC showed fast disintegration and the drug was released within 10–15 min in both media (Figure 3). The dissolution rate from HPC and HPMC mini-tablets were slower, with 80%–90% of the drug released after 60 min. The slowest release was seen for HPC mini-tablets. The formulations were found to behave similar in both media, suggesting that the formulations will not be influenced by the age-dependent pH level of the woman. This is beneficial in terms of developing a pharmaceutical product. It is of vital importance that the drug release characteristics are predictable and will show similar behavior in the population of potential users.

The somewhat faster release found from mini-tablets of HPMC as compared to HPC can be attributed to the less hydrophobic character of HPMC, and higher degree of swelling discussed above. It should be noted that the dissolution test can merely rank the dissolution rate from the different formulations, and not simulate vaginal *in vivo* conditions. As mentioned above, the amount of vaginal fluid present in the vagina at any time is less than 1 g on average [4]. Also, agitation is expected to be much lower than that simulated in the dissolution apparatus. Therefore, the release must also be expected to be slower *in vivo* than indicated by this simple *in vitro* test. This is why the bioadhesive characteristics of the multiparticulates are of great importance to maximize the effect of the drug.

### 3.4. Further Optimization

**Figure 4:**
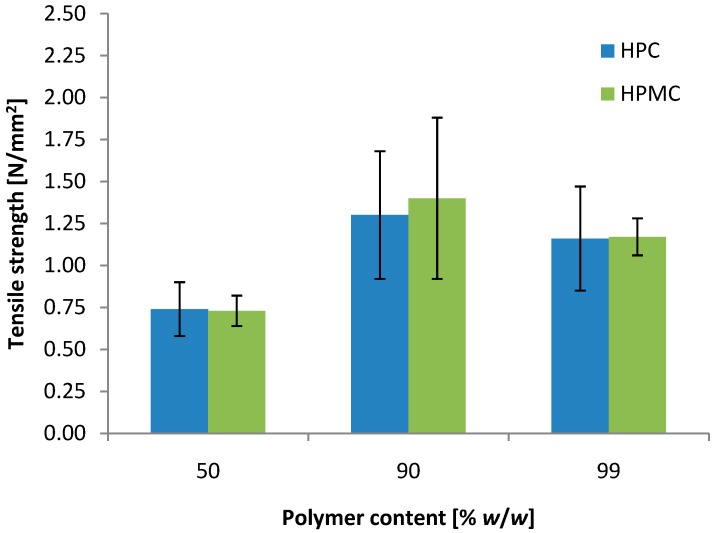
Effect of the polymer content on the tensile strength of 2 mm convex mini-tablets of HAL and polymer (*n* = 30).

Both mini-tablets based on HPC and HPMC seemed suitable for vaginal drug delivery, as they show bioadhesive nature towards vaginal tissue combined with a controlled release of the drug. Increasing the drug load in the mini-tablets leads to reduced amount of the matrix former. Additional mini-tablets of the ratio HAL to polymer 50:50 and 1:99 (*w*/*w*) were prepared to evaluate the effect of the drug load on the mechanical properties (Figure 4). As expected, the tensile strength of the tablets was reduced when the amount of polymer, working as dry binder, was reduced. However, at 50% (*w*/*w*) polymer content both HPC and HPMC were still able to produce appropriate mini-tablets with a tensile strength of approximately 0.75 N/mm^2^, which is adequate for handling related to packing, filling, transportation and use. No significant difference was found in the tensile strength by increasing the polymer content from 90% to 99% (*w*/*w*). It may be hypothesized that the available binding seats on particles of the 10:90 powder mixture are similar to that of the 1:99 powder mixture, resulting in tablets of similar mechanical strength. If the tensile strength increases with increasing polymer content due to increased fraction of the bond-forming material, it is likely that the tensile strength has reached a percolation threshold or a plateau around 90% (*w*/*w*) of polymer.

**Figure 5:**
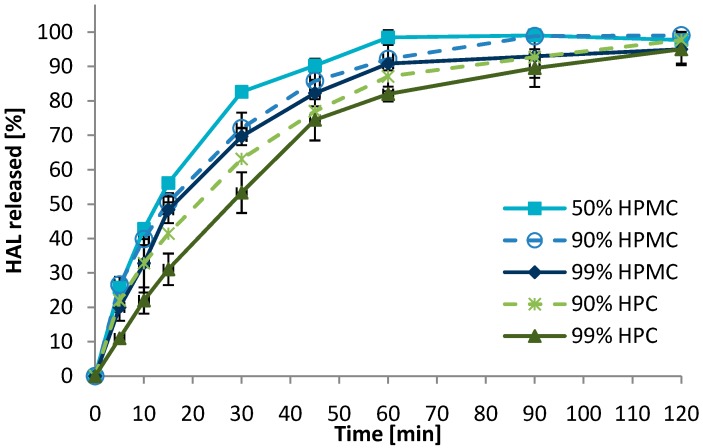
Effect of the polymer content (% *w*/*w*) on the dissolution rate of HAL from 2 mm convex mini-tablets (*n* = 3). Formulations containing 90% hydroxypropylmethyl cellulose (HPMC) and hydroxypropyl cellulose (HPC) (dashed lines) are replotted from Figure 3 to ease interpretation.

With respect to bioadhesion, no significant changes related to polymer content were observed in the detachment test. The dissolution rate of HAL was increased with reducing polymer content (Figure 5). The difference between 90% and 99% (*w*/*w*) polymer seemed larger for the more hydrophobic HPC as compared to the HPMC, where no significant difference was observed. However, reducing the polymer content to 50% resulted in an increased dissolution rate. Again, the dissolution test should merely be used to rank the formulations relative to each other, as the test conditions were not biorelevant with respect to amount of fluid and agitation.

In the current study, external lubrication with magnesium stearate in acetone was found to be sufficient, but for scale-up to industrial production, a lubricant needs to be included in the formulation. The less hydrophobic sodium stearyl fumarate seems as a suitable choice since it is less likely to influence the wetting and swelling of the polymers, as compared to the very hydrophobic magnesium stearate. Both formulations based on HPMC and HPC are assumed to possess suitable compression and compaction properties allowing scale up, however this remains to be demonstrated. Large scale production of mini-tablets could be regarded as challenging due to the small matrices potentially resulting in high variability, but products based on mini-tablets have been on the market for decades, (e.g., pancreatin products from Nordmark, Germany) proving that it is technologically feasible.

An approximation to a therapeutic dose of HAL for PDT in CIN (cervical cancer) may be derived from clinical studies where they applied 10 mL of 10 mM HAL thermogel topically [23,25] to be around 25 mg HAL. For mini-tablets with a mass of 5.5 ± 5 mg per unit and a drug load of 10% (*w*/*w*), this corresponds to around 45 mini-tablet, or 250 mg mini-tablets. This is considered to be a feasible amount to administer in one application, yet entailing enough single units to maintain the advantages of multiparticulates. Therefore, mini-tablet formulations of HAL in 10:90 (*w*/*w*) with HPMC and HPC seem to be promising candidates for further *in vivo* studies.

For administration, the new mini-tablet formulations might be filled into capsules, but clinical studies with volunteers have shown lack of correlation between *in vitro* and *in vivo* disintegration times for capsules, both with respect to hard gelatin and HPMC capsules, probably due to the low amount of vaginal fluid present [18,19]. Intravaginal disintegration times of more than 6 h were reported in several of the volunteers. Using an applicator that does not require packing of the multiparticulates into capsules, as suggested by Poelvoorde *et al.* [19], seems like a suitable way to eliminate the influence of capsule disintegration and deliver the mini-tablets intravaginally. It is anticipated that due to the small size, the mini-tablets will distribute evenly over the vaginal epithelium, and the bioadhesive nature will make them less sensitive to gravity and clearance, resulting in a longer residence time maximizing the effect of the drug. This still remains to be proven in *in vivo* studies.

From a safety perspective, the irritation potential of new vaginal delivery systems is the most vital characteristics because irritation causes inflammation and can, in severe cases, lead to cell toxicity and tissue damage, which further may increase the transmission of diseases (e.g., [37]). Also, mild mucosal irritation, such as genital burning, itching and discharge, is highly undesired for these types of products. Non-ionic cellulose derivatives in contrast to ionic polymers are not expected to influence the local pH nor form ionic bonds with the mucosal surface. Hydroxyethylcellulose (HEC) is even used as the base of the “universal placebo” gel that is commonly used in efficacy and safety trials of new microbicides [38,39]. The “universal placebo” gel is an isotonic get that is proven not to cause mucosal irritation in the standard rabbit vaginal irritation test assay [38], in the slug mucosal irritation assay [40] as well as in humans [39]. Whereas tonicity has been shown to be important to prevent epithelial swelling or dehydration [40], dry formulations seems to be less irritating on the vaginal mucosa [15,16,17,18], unless they contain materials that are irritating as such. This is supported by the several vaginal tablet products on the market. It is therefore assumed that mini-tablets based on non-ionic cellulose ester will be non-irritating and safe, but this remains to be verified in clinical studies.

## 4. Conclusions

Bioadhesive mini-tablets offer potential for improved residence time in the vaginal cavity targeting contact with mucosal tissue and prolonged release of the drug. Mini-tablets with a matrix of either HPMC or HPC were found to possess adequate mechanical strength, bioadhesive behavior towards cow vaginal tissue, and show pH independent controlled release of the drug, suggesting that both systems are equally suited for the treatment of both pre- and post-menopausal women. Mini-tablet formulations based on MC or HEC were mechanically weaker and disintegrated fast upon contact with fluids, and therefore released the full drug load within a few minutes. Bioadhesion towards vaginal tissue could not be successfully evaluated, either in the rotating cylinder test or in the detachment test.
